# Microbiomic signatures of anal fistula and putative sources of microbes

**DOI:** 10.3389/fcimb.2024.1332490

**Published:** 2024-01-19

**Authors:** Jun Yang, Ling Li, Wenya Su, Shuqin Zhang, Hai Xu, Mingyu Wang, Wenlong Shen

**Affiliations:** ^1^ Department of Anorectal Surgery, Qilu Hospital of Shandong University (Qingdao), Qingdao, Shandong, China; ^2^ State Key Laboratory of Microbial Technology, Microbial Technology Institute, Shandong University, Qingdao, China; ^3^ Department of Anorectal Surgery, Yinan Hospital of Traditional Chinese Medicine, Linyi, Shandong, China

**Keywords:** anal fistula, microbiome, source of microbes, 16S rDNA amplicon sequencing, microbiomic signatures of anal fistula

## Abstract

Anal fistula is a common perianal disease that typically develops from an abscess caused by in-flammation in the area. It has long been believed that intestinal microbes play a significant role in its development, considering its close relation to the intestinal environment. This work attempts to identify the microbiomic signatures of anal fistula, and putative sources of microbes by analyzing microbiomes of 7 anal fistula-associated sites in 12 patients. This study found that microbes in anal fistulas may originate from the skin surface in addition to the intestinal tract. This finding was further validated by NMDS analysis, which also indicated that the microbial communities in the inner and outer openings of the fistula were more similar to their surrounding environments. Using MaAslin2, the characteristics of the microbiome were examined, demonstrating a higher similarity between the abundant bacteria in the anal fistula samples and those found on the skin surface. Moreover, pin-to-pair analysis conducted on all subjects consistently showed a higher abundance of skin-sourced bacteria in anal fistulas. This study identifies the microbiomic signatures of anal fistula, and provides novel insights into the origin of microorganisms in anal fistulas.

## Introduction

Anal fistula is a common perianal disease that refers to an abnormal infectious fistula of the skin around the anal canal and rectum. The typical clinical manifestations encompass severe pain, perianal swelling, bleeding, and purulent discharge, significantly impeding patients’ social interaction, intimate relationships, and occupational engagements (de San Ildefonso Pereira et al., 2002). In general, men have a prevalence of 12.3 cases per 100,000 population, while women have a prevalence of 5.6 cases per 100,000 population (Sainio, 1984). In China, the incidence of anal fistula accounts for 1.67% - 3.6% of the total number of anorectal diseases. The peak age of the incidence is 20-40 years old, and the ratio of male to female is 5:1 to 6:1 (Dowling Enez and Izarra Henriquez, 2020). Surgical management of anal fistula, including anal fistulotomy, is effective for managing most simple and complex anal fistula. Although the control and eradication of anal fistula has achieved significant advancements, pathogenesis of anal fistula remains controversial.

Nowadays, the cryptoglandular theory is a widely accepted theory for the origin of anal fistula, suggesting that approximately 90-95% of anal fistula arise from anorectal abscesses (Parks, 1961; Sainio, 1984). Through culturing pus from patients with anorectal abscesses, both Eykyn and Grace (Eykyn and Grace, 1986) and Toyonaga et al. (Toyonaga et al., 2007) found that *Escherichia coli* and gut-specific *Bacteroides* are the absolute dominant flora in cultured flora in patients with fistula, comparing to patients without fistula. Similarly, many intestinal or skin microbiota, including biofilm-producing *E. coli*, *Enterococcus* spp., *Bacteroides fragilis*, *Staphylococcus aureus*, *Klebsiella pneumoniae*, *Prevotella* spp. and *Streptococcus viridans*, were isolated from stool samples or anorectal tissue samples in different perianal disease, such as inflammatory bowel disease (IBD), Crohn’s disease (CD), anorectal abscess and anal fistula (Tozer et al., 2015; Sugrue et al., 2017; Dowling Enez and Izarra Henriquez, 2020; Jaiswal et al., 2021; Breton et al., 2022). In general, the infection of gut-derived microorganisms was shown to be closely related to the formation of anal fistula (Toyonaga et al., 2007; van Onkelen et al., 2013; Leach et al., 2021).

However, previous research on the microbiota of anal fistula employed traditional culture methods, featured limited sample sizes, and exhibited various drawbacks (Seow-Choen et al., 1992; de San Ildefonso Pereira et al., 2002; Leach et al., 2021). The advancement of sequencing technology, such as 16S rDNA sequencing, allows for the identification of more abundant and difficult-to-culture bacteria than traditional microbial culture, resulting in a more effective methodology for microbiome research (Johnson et al., 2019; Zhang et al., 2023). In the past few years, several studies have examined the involvement of the gut microbiota in anal fistula by utilizing 16S rDNA sequencing and employing different experimental and control groups (van Onkelen et al., 2013; Tozer et al., 2015; Haac et al., 2019; Qiu et al., 2022; Cai et al., 2023). For example, Haac et al. compared patients’ stool and fistula samples using 16S rDNA sequencing, and concluded that *Achromobacter* and *Corynebacterium* were present at significantly higher levels in fistula samples, whereas *Bifidobacterium* was present at significantly higher levels in fecal samples than fistula samples (Haac et al., 2019). In addition, both Qiu et al. (Qiu et al., 2022) and Cai et al. (Cai et al., 2023) recruited anal fistula patients and healthy individuals for their studies. 16S rDNA sequencing was used to test the microbiome samples extracted from the intestinal swab or feces. When compared to healthy controls, fecal samples from anal fistula patients had a higher abundance of *Prevotella* spp., *Megamonas*, and *Lachnospira*, but a lower abundance of *Proteobacteria* spp., *Enterococcus*, *Bacteroides*, and *Klebsiella* (Qiu et al., 2022). However, Cai et al. found that *Blautia*, *Faecalibacterium*, *Ruminococcus*, *Coprococcus*, *Bacteroides*, *Clostridium*, *Megamonas* and *Anaerotruncus* were highly enriched in anal fistula patients, while the microbiome of healthy individuals was enriched with *Peptoniphilus* and *Corynebacterium* (Cai et al., 2023). Besides, there are other researchers collecting samples of preoperatively, immediately following surgery, 6-8 weeks postoperatively and at the time of any fistula recurrence in patients with rectovaginal fistula, in order to explore microbial taxa associated with recurrence. Their results showed that 31 taxa, including Bacteroidetes, *Alistipes* and *Rikenellaceae*, were enriched in patients undergoing successful repair (Leach et al., 2021). It was agreed that microbial compositions of anorectal fistulae samples were distinct from that in stool samples or healthy individuals. However, these studies have yielded inconsistent findings.

In this work, a more detailed investigation on the microbiomes of anal fistula and putative origins of microbes was performed by 16S rDNA amplicon high-throughput sequencing, with samples from seven different sites of same individuals. We collected the samples of the outer opening of anal fistula, healthy buttock skin on the opposite side of the outer opening of anal fistula, anal skin, inner opening of anal fistula, healthy anal gland on the opposite side of the inner opening of anal fistula, feces, and dissected anal fistula in patients with anal fistula. The microbial compositions and characteristics of different sites were analyzed to allow comparison of microbiomic signatures of different sites, and more importantly, to identify sources of microbes in anal fistula. This is a novel approach in the investigations on anal fistula, which led to novel findings as shown in this work.

## Materials and methods

### Sample acquisition

All the samples were collected between March and September 2023. Swabs were used to take samples from different sites of patients who came to Qilu Hospital of Shandong University (Qingdao) for anal fistula surgery.

For each participant, a sterile swab was used to swab 1) the outer opening of anal fistula; 2) healthy buttock skin on the opposite side of the outer opening of anal fistula; 3) anal skin; 4) inner opening of anal fistula; 5) healthy anal gland on the opposite side of the inner opening of anal fistula; 6) feces; 7) dissected anal fistula and then stored in sterile 10% glycerol solution and transported to the laboratory at low temperature (4°C).

### DNA extraction and 16S rDNA amplicon sequencing

Total DNA extractions of samples were performed using the DNeasy® PowerSoil® Kit (Qiagen, Germany) according to the type of sample and the manufacturer’s recommendations. 16S rDNA profiling targeting the V3-V4 hypervariable region was performed (341F: 5’-CCTACGGGNGGCWGCAG-3’, 805R: 5’-GACTACHVGGGTATCTAATCC-3’). The obtained fragments were purified by VAHTS DNA Clean Beads (Vazyme Biotech Co.,Ltd., Nanjing.,China). Using VAHTS® Universal DNA Library Prep Kit for IlluminaV3, VAHTS DNA Adapters set3 - set6 for Illumina (Vazyme Biotech Co.,Ltd., Nanjing.,China) for library building. Sequencing was performed with Illumina Novaseq 6000 platform under PE250 mode. The primer sequences were removed using QIIME2 v2021.4.0 (Bokulich et al., 2018). QIIME2 plugin vsearch (with default sequence similarity of 97%), Operational Taxonomic Units (OTU) classification was determined in Phylum, Genus and Species level respectively. Reference alignment and taxonomic assignments were based on the SILVA database (v138.1).

### Data analysis and statistics

Weighted Unifrac distance was calculated with phyloseq package on R platform. Hierarchal clustering was performed using the UPGMA method. MaAsLin2 (Microbiome Multivariable Association with Linear Models version 2.0) was used to identify biomarkers of microbiomes using linear models. ANOSIM and NMDS was performed with the vegan package on R platform. All statistics were performed with Jamovi 2.3.21.0 or Prism 9.4.1(681).

## Results and discussion

### Sample collection and sequencing

**Figure 1:**
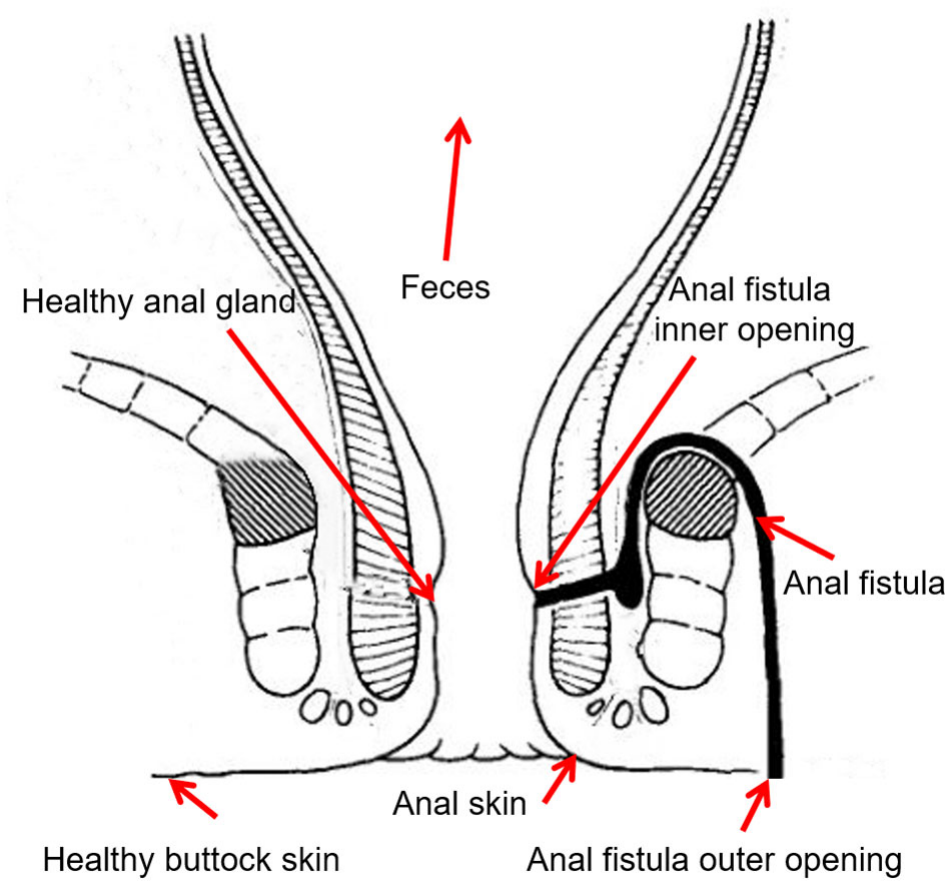
Sampling locations.

In order to study the microbiomic properties of anal fistula, and identify the origin of microbes in anal fistula, samples were collected from 12 patients suffering from anal fistula. Seven samples were collected from each patient including (**Figure 1**): 1) skin swab samples from the outer opening of anal fistula; 2) healthy buttock skin swab samples on the opposite side of the outer opening of anal fistula; 3) anal skin samples; 4) surface swab samples from the inner opening of anal fistula; 5) swab samples of healthy anal gland on the opposite side of the inner opening of anal fistula; 6) feces samples; 7) samples from dissected anal fistula. These samples represent possible sources of microbes in anal fistula. Subjects from whom samples were collected include 10 male, 2 female, with an average age of 43 years old. Second-generation 16S rDNA amplicon sequencing was performed on these samples to find out the microbiomic compositions of these samples.

The sampling locations from each patient were carefully designed. The inner opening, outer opening, and the inside of anal fistula were taken to represent the microbiomes associated with the pathogenic structure. The healthy anal gland that’s directly opposite to the inner opening of anal fistula represents a background control for the inner opening, and the healthy buttock skin that’s directly opposite to the outer opening of anal fistula represents a background control for the outer opening. In order to probe the putative origin of anal fistula microbes, fecal samples were also taken to represent the most likely origin of microbes from anal fistula. Anal skin samples were taken to represent the boundary of anal canal and buttock skin.

### Microbiomic compositions of collected samples and proposed origins of microbes from the anal fistula

**Figure 2:**
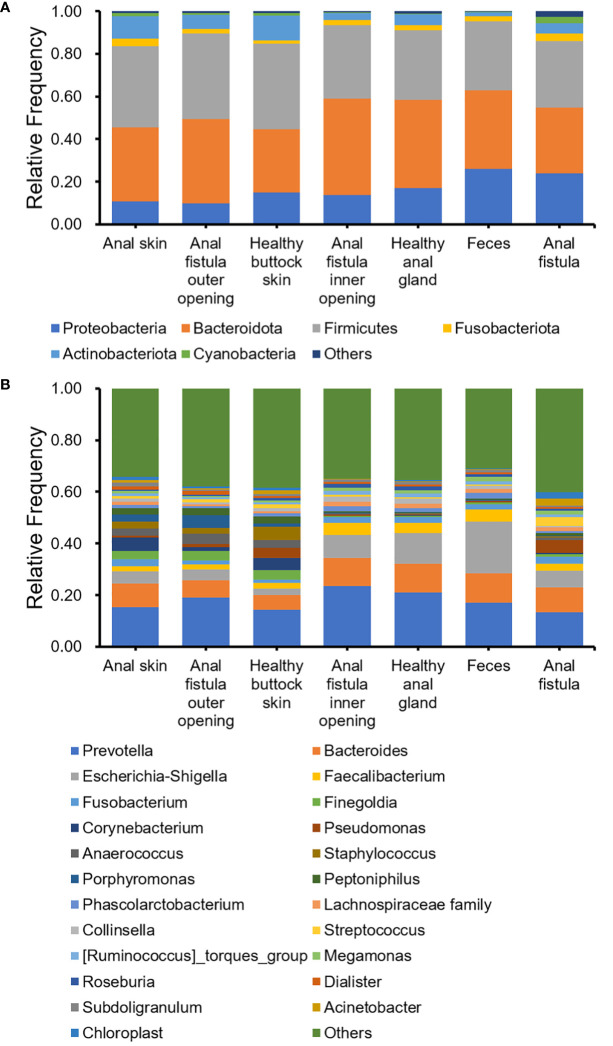
Bacterial compositions of collected samples. **(A)** Phylum level; **(B)** Genus level.

The 16S amplicon sequencing and subsequent annotation determined the microbiomic compositions of collected samples (**Figure 2**; **Supplementary Figures S1**, **S2**). Similarly to most other samples associated with human gastrointestinal tract, the majority of bacteria from the collected samples belong to Proteobacteria, Bacteroides, Firmicutes, and Actinobacteria phyla (**Figure 2A**). On the genus level, *Prevotella*, *Bacteroides*, *Escherichia-Shigella*, and *Faecalibacterium* are the most abundant genera (**Figure 2B**). Little difference was found on α-diversities when tested with paired *t*-tests (**Table 1**). Unlike on the phylum level, microbiomic compositions on the genus level showed a higher level of variability. This has prompted us to further look into the relations between microbiomes from different samples.

**Table 1:** Alpha diversities of microbiomes from different sampling locations.

Diversity	Anal skin	Anal fistula outer opening	Healthy buttock skin	Anal fistula inner opening	Healthy anal gland	Feces	Anal fistula
Chao1	199.73 ± 60.12	174.30 ± 36.45	225.71 ± 37.78	175.38 ± 58.89	228.42 ± 184.71	147.60 ± 41.63	176.73 ± 56.61^*^
ACE	199.88 ± 60.23	174.52 ± 36.50	225.79 ± 37.83	175.61 ± 58.98	228.75 ± 185.52	147.74 ± 41.63	176.93 ± 56.57^*^
Shannon	4.55 ± 0.61	4.55 ± 0.47	4.68 ± 0.59	4.16 ± 0.69	4.18 ± 1.00	3.68 ± 1.20	4.43 ± 0.93
Simpson	0.90 ± 0.06	0.91 ± 0.05	0.90 ± 0.04	0.86 ± 0.07	0.86 ± 0.08	0.79 ± 0.18	0.87 ± 0.10

*, statistically different from healthy buttock skin.

**Figure 3:**
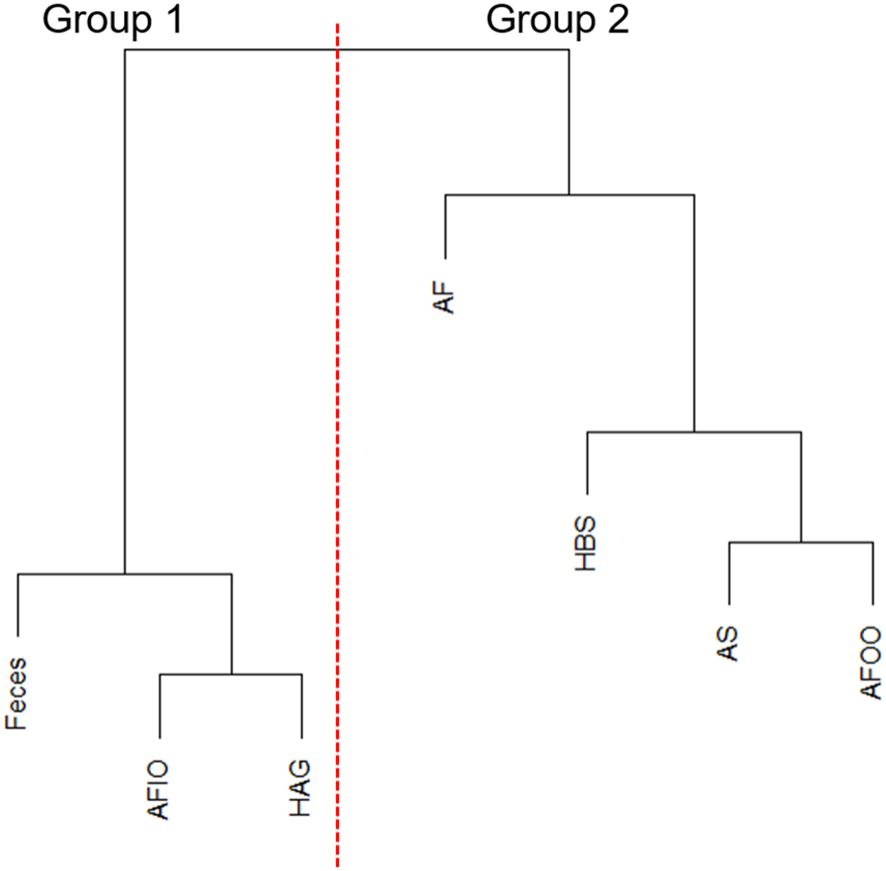
Hierarchal clustering of microbiomes. AFIO, anal fistula inner opening; HAG, healthy anal gland; AF, anal fistula; HBS, healthy buttock skin; AS, anal skin; AFOO, anal fistula outer opening.

With hierarchal clustering of average microbiomes from anal fistula (AF), feces, anal fistula outer opening (AFOO), anal fistula inner opening (AFIO), anal skin (AS), healthy anal gland (HAG), and healthy buttock skin (HBS) using weighted UniFrac distances, it can be observed that these microbiomes are clearly clustered into two distinct groups (**Figure 3**). Samples from within the anal canal (feces, anal fistula inner opening, and healthy anal gland) are very similar in compositions and form a cluster (Group 1), and samples from the buttock including healthy buttock skin, anal skin, and anal fistula outer opening are very similar in bacterial compositions and form a second cluster (Group 2). Intriguingly, samples from anal fistula can be clustered with Group 2, and showed similar bacterial compositions with skin-originated samples. This has prompted us to propose that bacteria from anal fistula may originate from the buttock skin in addition to the feces, and that infections originated from the skin may also be responsible for the development of anal fistula.

**Figure 4:**
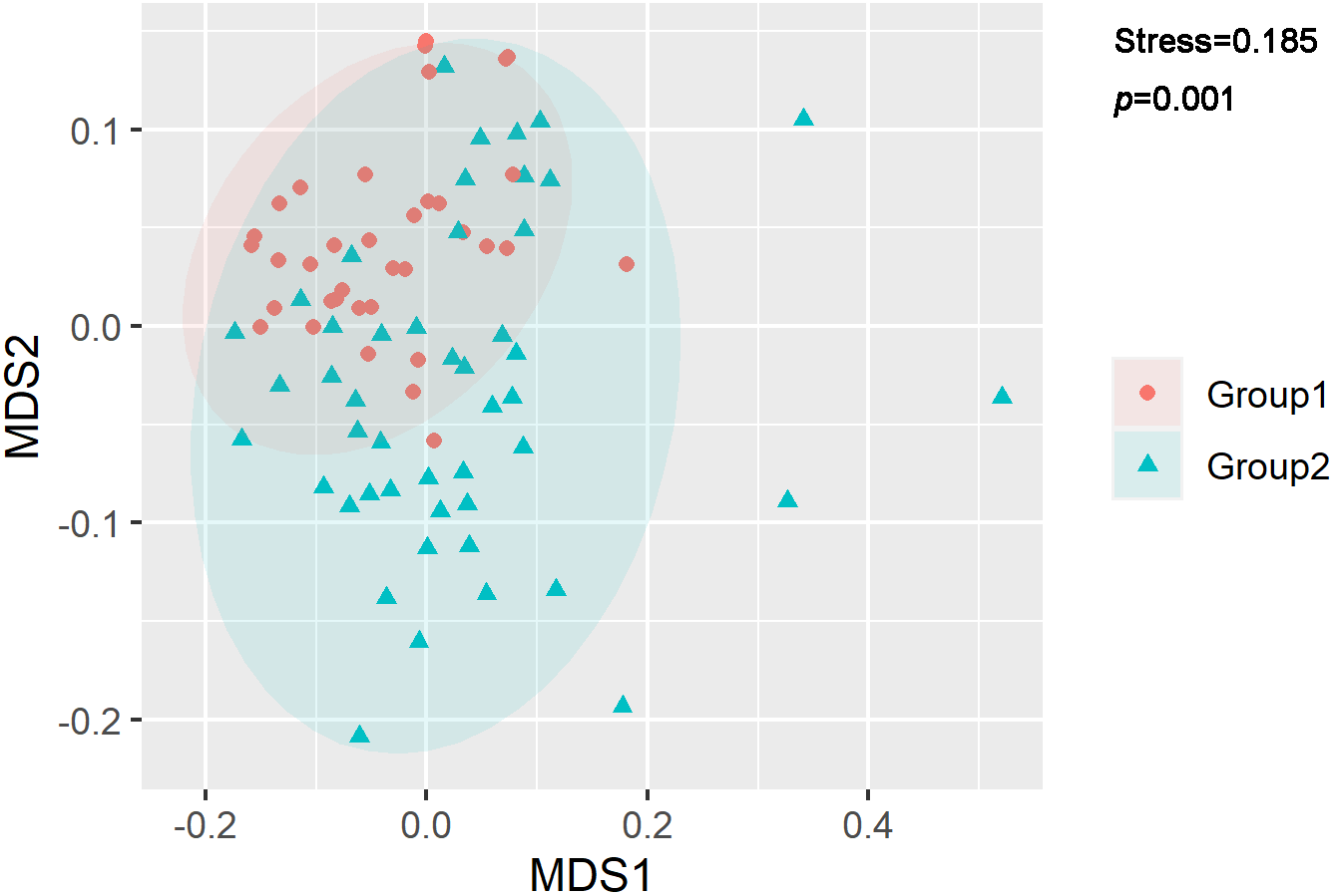
NMDS analysis of microbiomes. *p*-values were calculated with ANOSIM.

Further NMDS analysis supports this proposal. As shown in **Figure 4**, microbiomes from Group 1 and Group 2 can be statistically distinguished (*p*=0.001, ANOSIM), suggesting when anal fistula microbiomes are clustered with other skin-originated microbiomes, significant differences can be found from anal canal-originated microbiomes.

### Microbiomic signatures of anal fistula and related microbiomes

MaAsLin2 is a linear model-based method for identification of biomarkers from microbiomes, and was considered to cause fewer false positives comparing with its counterpart LefSe (Mallick et al., 2021). This algorithm was therefore used to identify microbiomic signatures of the microbiomes studied in this work. With a FDR cutoff of 0.1, we found 15 taxa that showed significant increase or decrease between Group 1 and Group 2 microbiomes (**Table 2**). Group 1 microbiomes, aka the anal canal-related microbiomes were enriched with *Faecalibacterium*, *Escherichia-Shigella*, *Prevotella* (but not *Prevotella timonensis*), and *Lachnospiraceae* species, that are common gut microbiome-related microbes. Group 2 microbiomes, aka the buttock skin-associated microbiomes and anal fistula microbiomes, were enriched with *Anaerococcus*, *Finegoldia*, *P. timonensis*, *Peptoniphilus* species, and *Negativicoccus*.

**Table 2:** Microbiomic biomarkers of Group 1 and Group 2 samples.

Biomarker	Group 1 abundance	Group 2 abundance	Fold difference	FDR
More abundant in Group 1				
***Faecalibacterium***	0.050	0.028	1.82	0.0732
***Lachnospiraceae_UCG-010* sp.**	2.13×10^-3^	6.87×10^-4^	3.10	0.0732
***GCA-900066575***	1.84×10^-4^	4.97×10^-5^	3.69	0.0732
***Lachnospiraceae_UCG-010***	1.92×10^-4^	4.16×10^-5^	4.63	0.0732
***Escherichia*-*Shigella***	0.156	0.054	2.92	0.0860
***Prevotella***	0.206	0.114	1.80	0.0883
More abundant in Group 2				
***Anaerococcus***	1.41×10^-3^	0.018	12.5	0.0319
***Finegoldia***	7.32×10^-3^	0.035	4.80	0.0732
***Peptoniphilus***	3.40×10^-3^	0.020	5.75	0.0732
***Prevotella timonensis***	6.60×10^-4^	7.21×10^-3^	10.9	0.0732
***Peptoniphilus lacrimalis***	3.86×10^-4^	2.24×10^-3^	5.81	0.0860
***Peptoniphilus coxli***	7.27×10^-4^	3.36×10^-3^	4.63	0.0860
***Negativicoccus***	1.01×10^-4^	1.72×10^-3^	17.0	0.0860
***Peptoniphilaceae* sp.**	2.63×10^-6^	2.22×10^-4^	84.5	0.0860
***Peptoniphilus obesi***	9.66×10^-6^	2.37×10^-4^	24.5	0.0951

Comparison was made with MaAsLin2.

Comparison between Group 2 microbiomes with anal fistula microbiomes suggested that only *Rothia* was found to be enriched in anal fistula samples (**Supplementary Figure S3**). This is in agreement with our suggestion that anal fistula microbiomes are clustered with Group 2 microbiomes, as only few differences can be found between anal fistula and Group 2 microbiomes. Comparison between Group 1 microbiomes with anal fistula microbiomes, on the other hand, led to the finding of 20 genera enriched in anal fistula (**Table 3**), again confirming that Group 1 microbiomes and anal fistula microbiomes are significantly different. Interestingly, *Rothia* was also enriched in anal fistula microbiomes. A further inspection of the abundance of *Rothia* showed that it has an average relative abundance of 1.15×10^-4^ in anal fistula, significantly higher than in Group 1 microbiomes (average 1.34×10^-5^, *p*=6.74×10^-5^, two-tailed *t*-test) and Group 2 microbiomes (average 1.92×10^-5^, *p*=6.71×10^-4^, two-tailed *t-*test). *Rothia* is a human pathogen that has been implicated in many infections and the development of periodontal disease (Fatahi-Bafghi, 2021). The higher *Rothia* abundance in anal fistula is an implication that this genus may play a role in the development of anal fistula. This, however, does not suggest that *Rothia*’s role in anal fistula is concluded, as this study only reports associational results that still require mechanistic and etiological investigations for make such a strong claim.

**Table 3:** Microbiomic biomarkers of anal fistula comparing with Group 1 samples.

Biomarker	Anal fistula abundance	Group 1 abundance	Fold difference	FDR
More abundant in anal fistula				
***Enhydrobacter***	5.73×10^-4^	2.65×10^-5^	21.6	7.95×10^-3^
***Janthinobacterium***	7.36×10^-4^	3.72×10^-5^	19.8	7.95×10^-3^
***Rothia***	1.15×10^-4^	1.34×10^-5^	8.58	7.95×10^-3^
***Sphingomonas***	8.90×10^-4^	8.19×10^-5^	10.9	0.011
***Acinetobacter guillouiae***	0.029	2.30×10^-3^	12.7	0.016
***Actinomyces***	1.70×10^-4^	2.07×10^-5^	8.21	0.018
***Delftia***	2.35×10^-4^	1.94×10^-5^	12.1	0.024
**Caulobacteraceae sp.**	4.25×10^-3^	2.10×10^-4^	20.2	0.026
***Mitochondria***	2.85×10^-4^	8.62×10^-6^	40.7	0.026
***Streptococcus anginosus***	0.012	6.50×10^-4^	18.0	0.035
***Stenotrophomonas***	2.66×10^-4^	1.41×10^-5^	18.8	0.035
***Bradyrhizobium***	1.21×10^-3^	5.34×10^-5^	22.7	0.039
***Pelomonas***	1.38×10^-3^	2.09×10^-4^	6.62	0.040
***Schaalia turicensis***	2.87×10^-3^	2.23×10^-4^	12.9	0.078
***Corynebacterium***	3.64×10^-3^	5.20×10^-4^	7.00	0.081
***Staphylococcus***	4.39×10^-3^	3.21×10^-4^	12.7	0.083
***Chryseobacterium***	2.20×10^-4^	7.78×10^-6^	28.3	0.083
***Anaerococcus***	5.84×10^-3^	1.41×10^-3^	4.14	0.085
***Muribaculaceae* sp.**	1.72×10^-3^	1.18×10^-3^	1.46	0.085
***Diaphorobacter***	1.34×10^-3^	1.26×10^-5^	106	0.088
More abundant in Group 1				
***Alloprevotella***	4.14×10^-3^	9.38×10^-3^	2.27	0.088

Comparison was made with MaAsLin2.

The large number of selectively enriched bacterial groups in anal fistula microbiomes *versus* Group 1 microbiomes prompted us to wonder whether these bacterial groups may originate from buttock skin-related microbiomes (Group 2 microbiomes). To verify this, the relative abundances of these bacterial groups were compared between Group 1 and Group 2 microbiomes (**Supplementary Figures S4**-**S15**). With pairwise comparison in each subject, we found that most of these bacterial groups are present in a higher abundance in Group 2 microbiomes. With a total of 252 comparisons, in 144 comparisons Group 2 microbiomes contain a higher abundance of these selectively enriched bacterial groups in anal fistula, while in only 26 comparisons Group 1 microbiomes contain a higher abundance. Particularly, for may bacterial groups, especially the highly abundant bacteria, such as *Corynebacterium* (12/12 samples), *Staphylococcus* (11/12 samples), *Anaerococcus vaginalis* (11/12 samples), *Acinetobacter guillouiae* (9/12 samples), *Streptococcus anginosus* (10/12 samples), *Sphingomonas* (9/12 samples), *Janthinobacterium* (9/12 samples), Group 2 microbiomes showed a higher abundance comparing with Group 1 microbiomes. These results strongly suggest that the enriched bacterial groups in anal fistula comparing with Group 1 microbiomes are also present with higher abundancies in Group 2 microbiomes, leading to the proposal that microbes in anal fistula may have a skin origin in certain scenarios. Indeed, in certain cases such as subject G22, anal fistula was populated with *Pseudomonas* at high abundance (60.00%), while this bacterium is present at low levels of intestine related samples: feces 0.08%, and healthy anal gland 0.09%. In contrary, in skin-related samples, *Pseudomonas* is an abundant bacterium: healthy buttock skin 6.96%, and anal fistula outer opening 3.75%. It is reasonable to propose that *Pseudomonas* in anal fistula originate from the skin, as it is essentially absent within normal intestinal tract.

### Proposal of origin of microbes in anal fistula

The clear and significant clustering of anal fistula microbiomes with buttock skin-related microbiomes instead of anal canal-related microbiomes is a surprise. The gastrointestinal tract is the largest and most prominent reservoir and source of bacteria in the human body. It is natural to hypothesize that any infection in the gastrointestinal tract is related to gut microbiota. Anal fistula, in many cases, are developed from perianal abscess which is essentially an infection. Therefore, we originally expected that microbiomes of anal fistula are more closely related to anal canal-related microbiomes. This surprising finding prompted us to further analyze the microbiomes measured in this work.

Another interesting observation is that the two openings of the anal fistula were also found to carry microbiomes that are similar to their surroundings: the inner opening to healthy anal gland, and the other opening to healthy buttock skin. It appears that anal fistula microbiomes do not impact the two openings.

Very few previous research studied the microbiomes of anal fistula. In one of the few studies, the authors found the enrichment of *Achromobacter* and *Corynebacterium* in anal fistula samples (Haac et al., 2019). The enrichment of *Corynebacterium* was also found in this work. There are even fewer investigations on where the microbes in anal fistula originates. Most of the work focused on the microbiomic compositions of fecal samples of anal fistula patients. This work presents a novel study comparing the microbiomes of different samples from anal fistula patients, with the goal of proposing the origin of microbes in anal fistula.

Unlike what we originally expected, this work led to the proposal that anal fistula microbes can originate from other sources in addition to the intestinal microbiota, including buttock skin. This is supported by several analyses, including the similar microbiomic composition of anal fistula with buttock skin-related samples, the similar increase of a set of bacterial groups in buttock skin-related samples and anal fistula samples *versus* anal canal-related samples, the apparent extra-gastrointestinal source of certain increased bacterial groups in anal fistula (*Staphylococcus* and *Anaerococcus vaginalis*, for instance), and the finding that in a subject the populating microbes in anal fistula are absent in the intestinal tract. All evidences point to the theory that microbial groups enriched in anal fistula-which are possibly what caused anal fistula- can also originate from buttock skin. Due to the limited sample sizes in this work, we would like to recommend more sophisticated studies to confirm this finding. Nevertheless, this proposal provides us with a likely surprising hypothesis for the origin of microbes in anal fistula.

## Conclusion

This study investigated the microbiomes of different samples from anal fistula patients, and compared their compositions. Our most prominent finding is that the microbiomes of anal fistula are closer to the buttock skin-related microbiomes than the anal canal-related microbiomes. Further comparison led to the proposal that the microbes in anal fistula can also originate from the buttock skin, in addition to the gastrointestinal tract. It is also observed that *Rothia* may play a role in the development of anal fistula. This work represents a detailed comparison of microbiomes from different samples of anal fistula patients, and provided evidence that led to the proposal of origins of microbes in anal fistula.

## Data availability statement

The datasets presented in this study can be found in online repositories. The names of the repository/repositories and accession number(s) can be found in the article/**Supplementary Material**.

## Ethics statement

The studies involving humans were approved by Medical Ethics Committee of Qilu Hospital of Shandong University (Qingdao) under approval number KYLL-2023045. The studies were conducted in accordance with the local legislation and institutional requirements. The participants provided their written informed consent to participate in this study. The study was conducted in accordance with the Declaration of Helsinki, and were approved by Medical Ethics Committee of Qilu Hospital of Shandong University (Qingdao) under approval number KYLL-2023045. The study was conducted in accordance with the local legislation and institutional requirements. Written informed consent was obtained from the individual(s) for the publication of any potentially identifiable images or data included in this article.

## Author contributions

JY: Writing – original draft. LL: Writing – original draft. WYS: Writing – original draft. SZ: Writing – original draft. HX: Writing – review & editing. MW: Funding acquisition, Writing – review & editing. WLS: Writing – review & editing.
