# Supplementary material for: Microbiomic signatures of anal fistula and putative sources of microbes
Source: Front Cell Infect Microbiol. 2024 Jan 19;14:1332490. doi: 10.3389/fcimb.2024.1332490 (PMC10834682; doi:10.3389/fcimb.2024.1332490)
Supplement: Supplementary file 1 [file Table_1.docx]

Supplementary Material

## Supplementary Figures

Figure S1.Bacterial compositions of collected samples on the phylum level

Figure S2. Bacterial compositions of collected samples on the genus level

Figure S3. Microbiomic biomarker of anal fistula *versus* Group 2 samples

Figure S4. Comparison of bacterial abundances between Group 1 and Group 2 samples in subject G12. Microbiomic biomarkers of anal fistula versus Group 1 samples were compared.

Figure S5. Comparison of bacterial abundances between Group 1 and Group 2 samples in subject G13. Microbiomic biomarkers of anal fistula versus Group 1 samples were compared.

Figure S6. Comparison of bacterial abundances between Group 1 and Group 2 samples in subject G19. Microbiomic biomarkers of anal fistula versus Group 1 samples were compared.

Figure S7. Comparison of bacterial abundances between Group 1 and Group 2 samples in subject G20. Microbiomic biomarkers of anal fistula versus Group 1 samples were compared.

Figure S8. Comparison of bacterial abundances between Group 1 and Group 2 samples in subject G21. Microbiomic biomarkers of anal fistula versus Group 1 samples were compared.

Figure S9. Comparison of bacterial abundances between Group 1 and Group 2 samples in subject G22. Microbiomic biomarkers of anal fistula versus Group 1 samples were compared.

Figure S10. Comparison of bacterial abundances between Group 1 and Group 2 samples in subject G23. Microbiomic biomarkers of anal fistula versus Group 1 samples were compared.

Figure S11. Comparison of bacterial abundances between Group 1 and Group 2 samples in subject G28. Microbiomic biomarkers of anal fistula versus Group 1 samples were compared.

Figure S12. Comparison of bacterial abundances between Group 1 and Group 2 samples in subject G41. Microbiomic biomarkers of anal fistula versus Group 1 samples were compared.

Figure S13. Comparison of bacterial abundances between Group 1 and Group 2 samples in subject G44. Microbiomic biomarkers of anal fistula versus Group 1 samples were compared.

Figure S14. Comparison of bacterial abundances between Group 1 and Group 2 samples in subject G45. Microbiomic biomarkers of anal fistula versus Group 1 samples were compared.

Figure S15. Comparison of bacterial abundances between Group 1 and Group 2 samples in subject G47. Microbiomic biomarkers of anal fistula versus Group 1 samples were compared.
