# Supplementary figures and images for: Microbiomic signatures of anal fistula and putative sources of microbes
Source: Front Cell Infect Microbiol. 2024 Jan 19;14:1332490. doi: 10.3389/fcimb.2024.1332490 (PMC10834682; doi:10.3389/fcimb.2024.1332490)

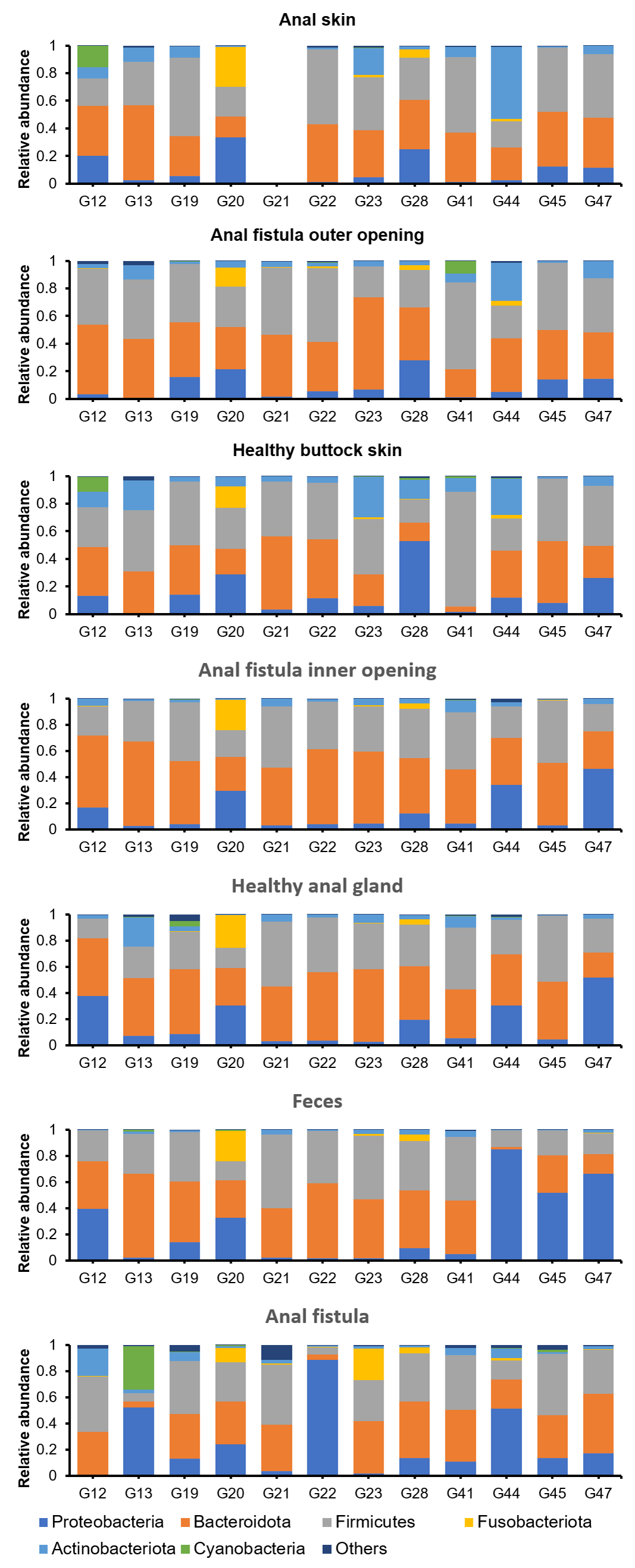

Supplement: Supplementary file 2 [file Image_1.tif]

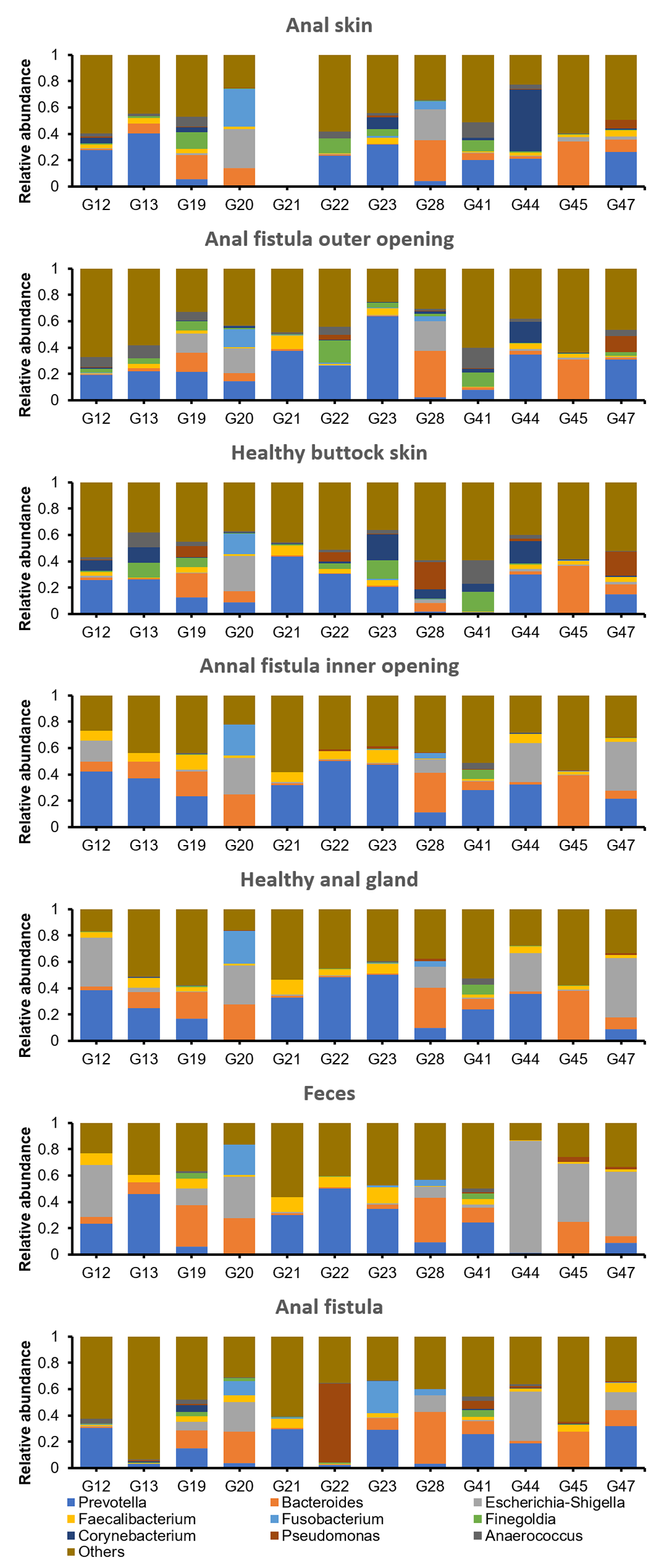

Supplement: Supplementary file 3 [file Image_2.tif]

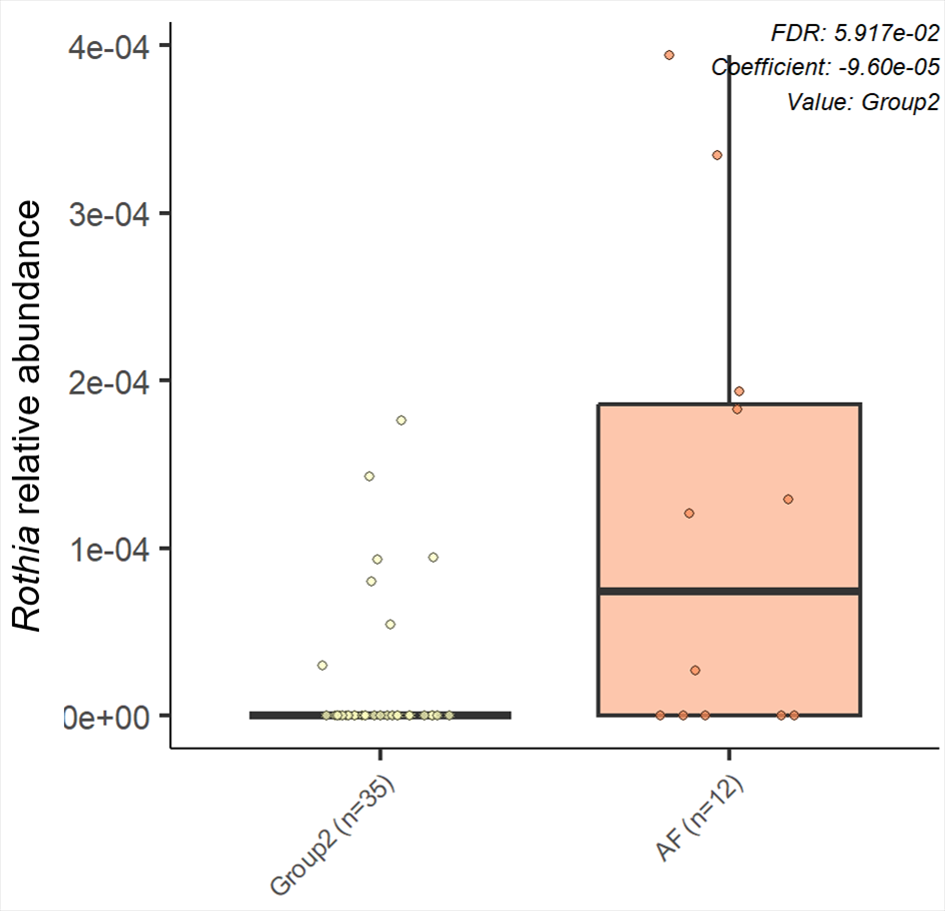

Supplement: Supplementary file 4 [file Image_3.tif]

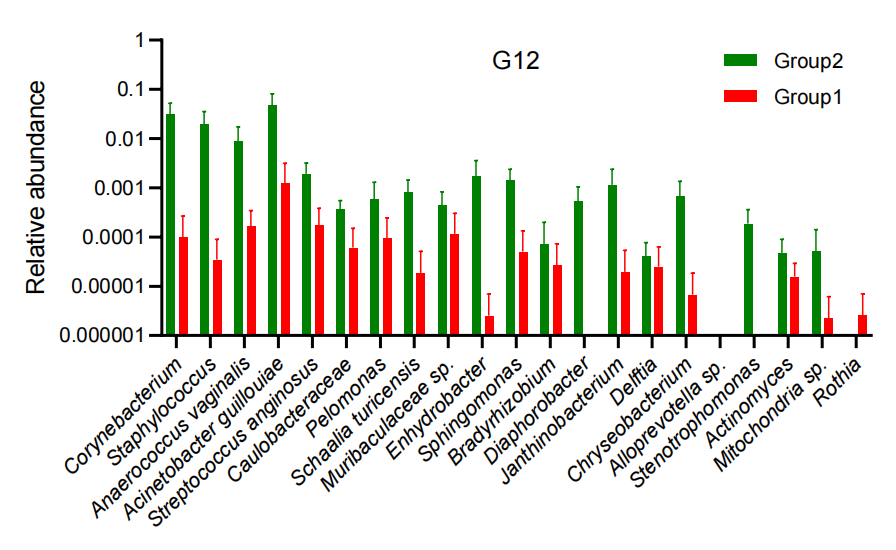

Supplement: Supplementary file 5 [file Image_4.tif]

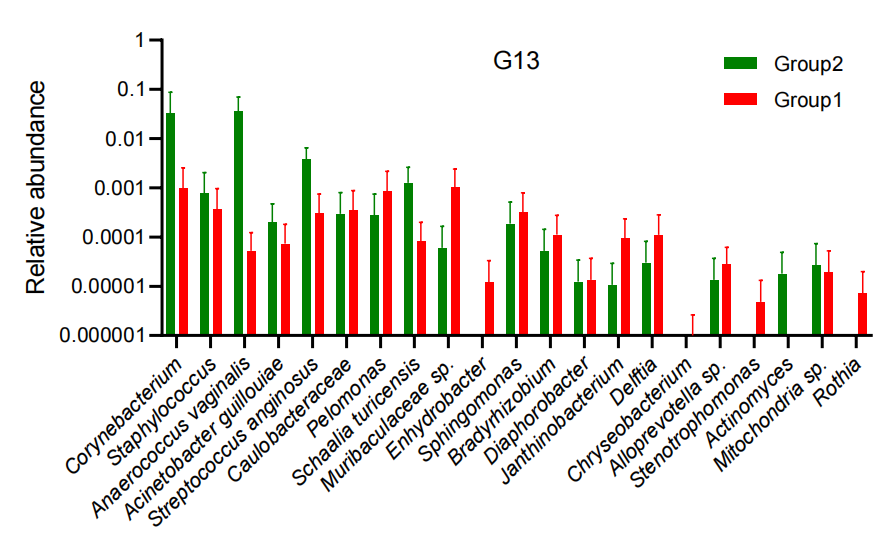

Supplement: Supplementary file 6 [file Image_5.tif]

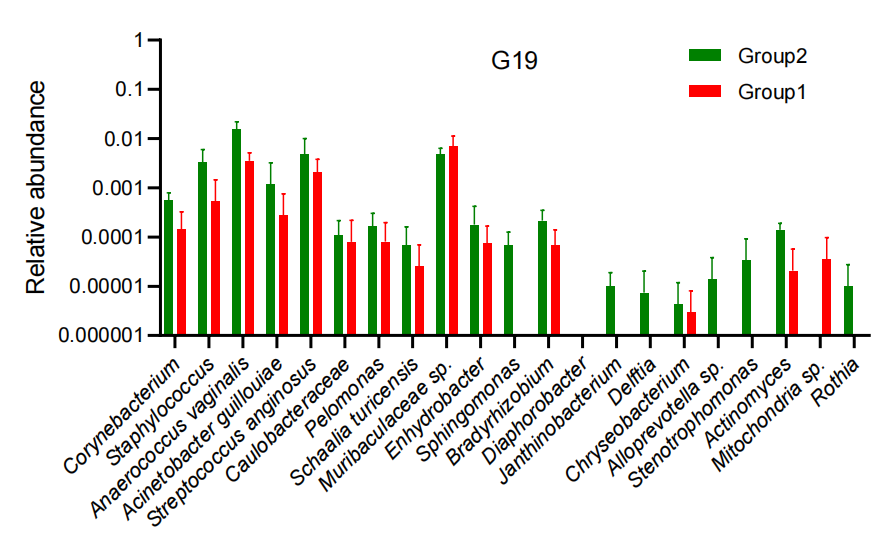

Supplement: Supplementary file 7 [file Image_6.tif]

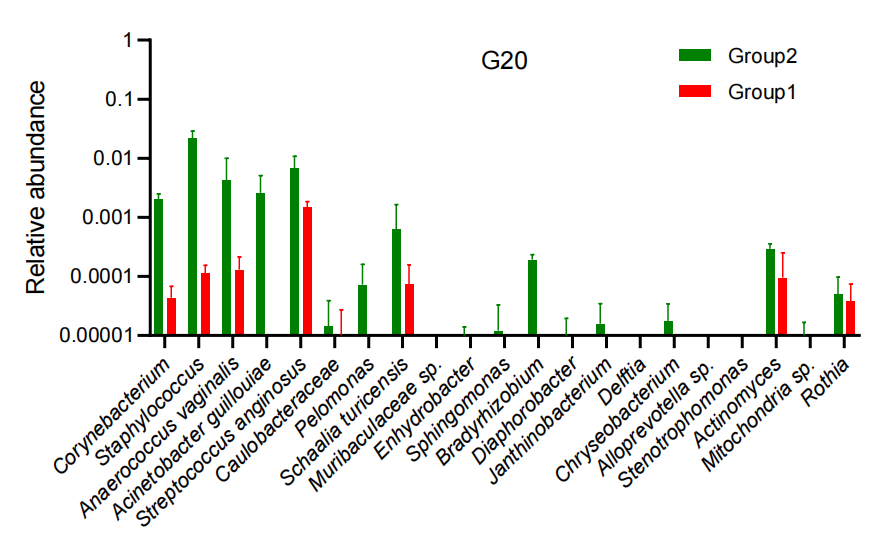

Supplement: Supplementary file 8 [file Image_7.tif]

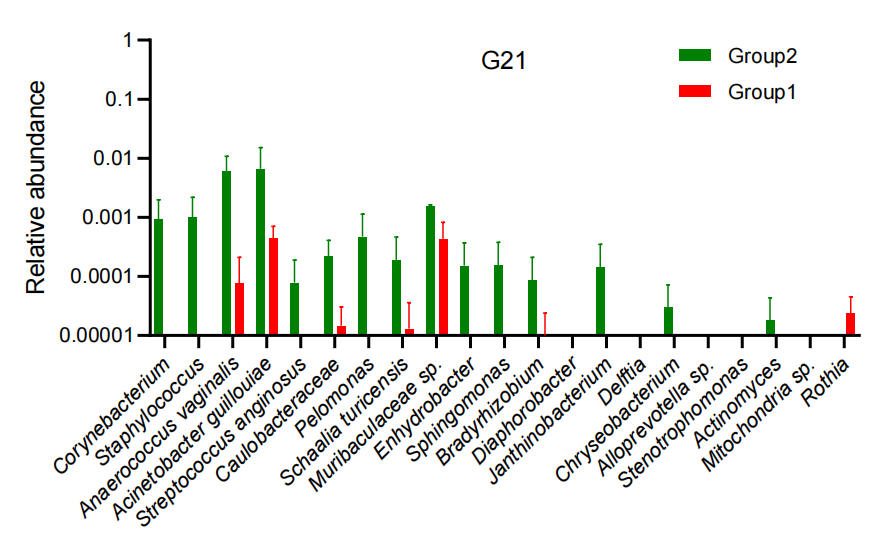

Supplement: Supplementary file 9 [file Image_8.tif]

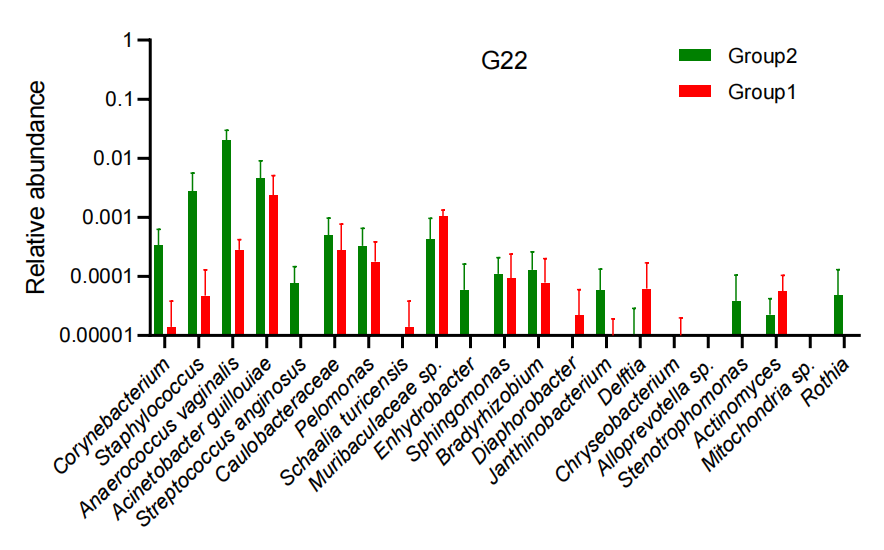

Supplement: Supplementary file 10 [file Image_9.tif]

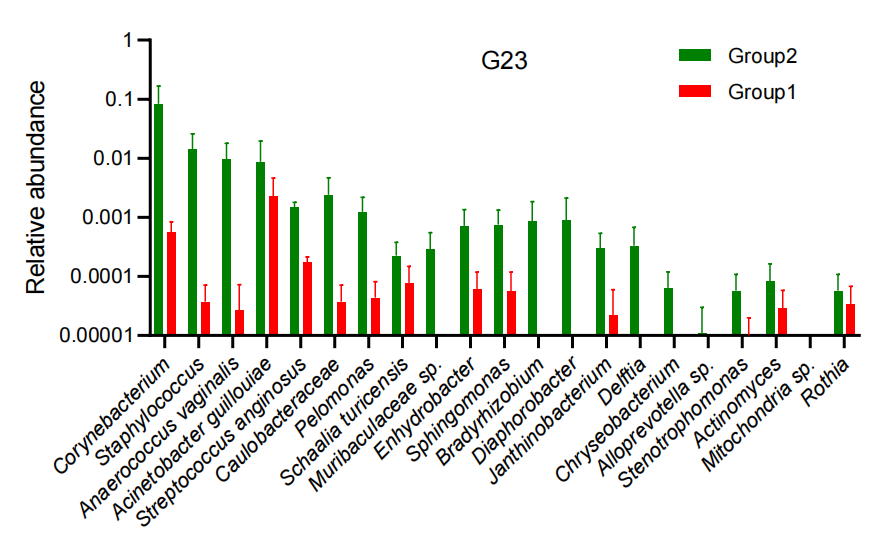

Supplement: Supplementary file 11 [file Image_10.tif]

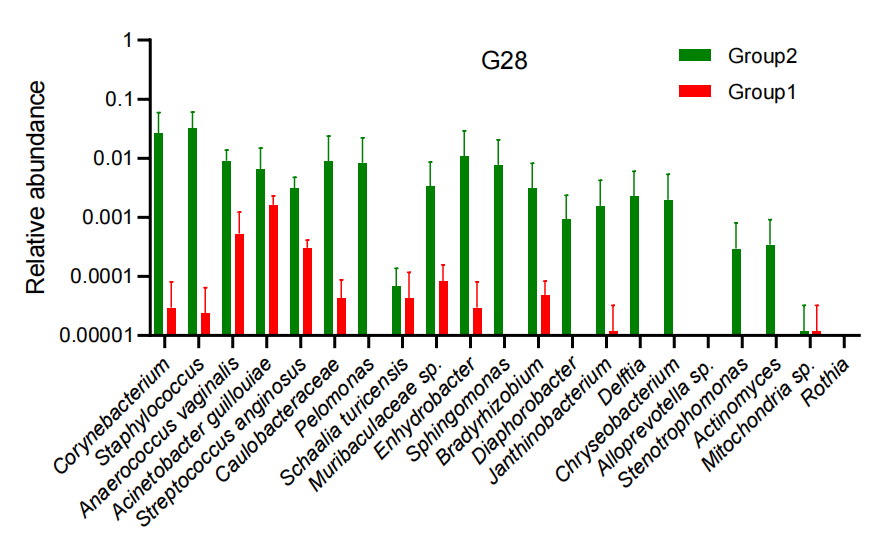

Supplement: Supplementary file 12 [file Image_11.tif]

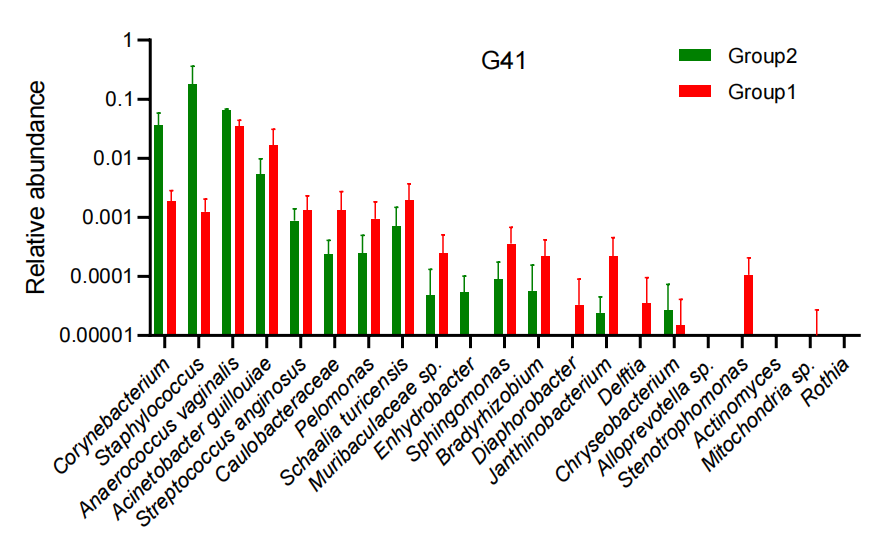

Supplement: Supplementary file 13 [file Image_12.tif]

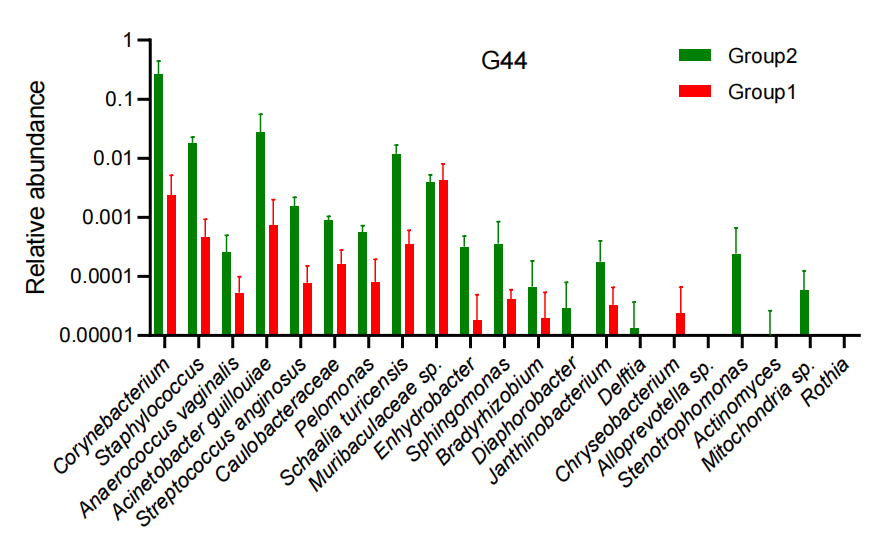

Supplement: Supplementary file 14 [file Image_13.tif]

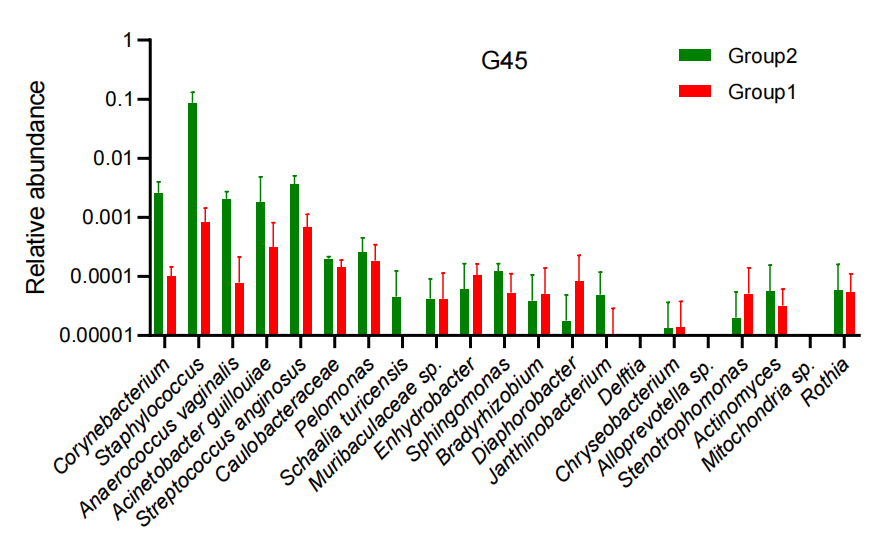

Supplement: Supplementary file 15 [file Image_14.tif]

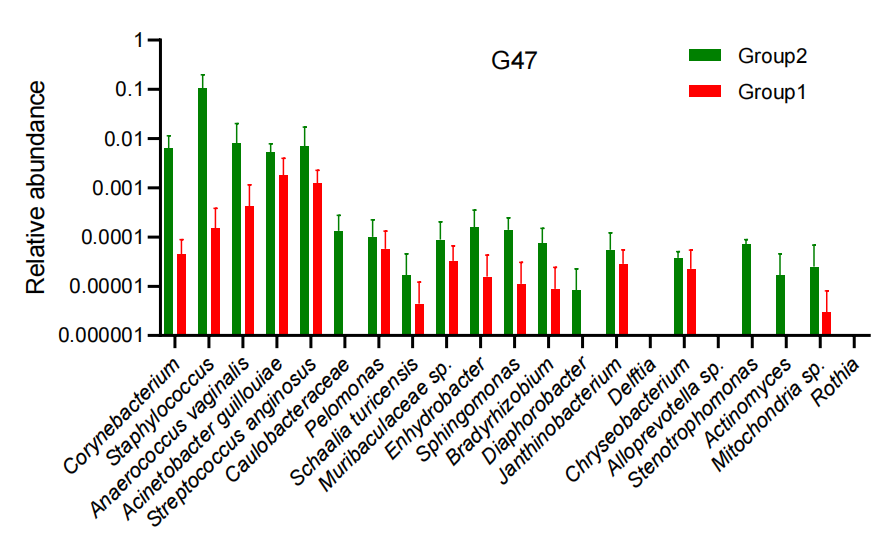

Supplement: Supplementary file 16 [file Image_15.tif]
